# Excessive waitlists and delays to treatment with low-dose-rate brachytherapy predict an increased risk of recurrence and metastases in intermediate-risk prostatic carcinoma

**DOI:** 10.1016/j.ctro.2021.06.008

**Published:** 2021-07-01

**Authors:** Rutvij A. Khanolkar, Harvey Quon, Kundan Thind, Michael Sia, Michael Roumeliotis, Siraj Husain, Philip McGeachy, Tyler Meyer, Kevin Martell

**Affiliations:** a Department of Oncology, University of Calgary, Calgary, AB, Canada; b Alberta Health Services, Calgary, AB, Canada

**Keywords:** Prostate cancer, Brachytherapy, Wait times for treatment, Recurrence, Metastases

## Abstract

**Purpose:**

It has previously been shown that increased wait times for prostatectomy are associated with poorer outcomes in intermediate-risk prostatic carcinoma (PCa). However, the impact of wait times on PCa outcomes following low-dose-rate brachytherapy (LDR-BT) are unknown.

**Methods and Materials:**

We retrospectively reviewed 466 intermediate-risk PCa patients that underwent LDR-BT at a single comprehensive cancer center between 2003 and 2016. Wait times were defined as the time from biopsy to LDR-BT. The association of wait times with outcomes was evaluated using Cox and Fine-Gray regression in both univariate and multivariate models.

**Results:**

Median (interquartile range) follow-up and wait time for all patients were 8.1 (6.3–10.4) years and 5.1 (3.9–6.9) months, respectively. Among NCCN unfavourable intermediate-risk (UIR) patients (n = 170; 36%), increased wait times predicted both a greater cumulative incidence of recurrence [MHR = 1.01/month of wait time (95% CI: 1.00–1.03); P = 0.044] and metastases [MHR = 1.04/month of wait time (95% CI: 1.02–1.06); P < 0.001] in multivariate modeling. In NCCN favourable intermediate-risk (FIR) patients, there was no significant association between wait time and recurrence or metastases risk. Among all intermediate-risk patients, wait time was associated with an increase in the incidence of metastases [MHR = 1.03/month of wait time (95% CI: 1.02–1.05); P < 0.001], but not recurrence in multivariate models. There was no association between wait time and overall survival in the UIR, FIR, or all intermediate-risk cohorts.

**Conclusions:**

Resource constraints within this center’s public healthcare system have contributed to waitlists exceeding 5-months in length. This study finds that patients with UIR PCa experience a 1% increase in the risk of recurrence and 4% increase in the risk of metastases with each additional month of delay in definitive disease management. Preventing such extended management delays in LDR-BT may improve disease-related outcomes in patients with PCa.

## Highlights

• Resource constraints have led to prolonged wait-times for prostate brachytherapy.
• Increased wait times predict a significant increase in recurrence and metastases.
• Better resource planning is needed to reduce management delays & improve outcomes.

## 1 Introduction

Prostate cancer (PCa) is one of the most commonly diagnosed malignant neoplasms in North America [1], [2]. The number of patients requiring treatment, and hence the overall economic burden of prostate cancer, is expected to increase over the next decade [3], [4], [5], [6]. This is important in resource constrained environments such as the Canadian health care system where an increased incidence of disease often leads to prolonged waitlists for treatment while infrastructure and resources are implemented.

These increased wait times could become a source of anxiety for patients and may also affect patient outcomes. For example, a previous study found that management delays in prostatectomy for intermediate-risk PCa were associated with worse recurrence-free survival and increased biochemical recurrence [7]. A similar, more recent study showed that longer wait times were associated with an increase in the UCSF-CARPA score, which have been previously been shown to be predict the risk of biochemical recurrence [8], [9]. Although some studies suggest short delays to treatment are safe, several other international studies have also found that delays in surgical treatment for PCa predict poorer outcomes in patients [10], [11], [12].

Unfortunately, the potential for impact of wait times to radiotherapy treatments for prostate cancer has not been explored as of yet. On review, this is likely due to most radiotherapy treatments being less dependent on hospital resources such as operating room staffing. At this tertiary study center, however, patients enrolled for treatment with low-dose-rate brachytherapy (LDR-BT) for intermediate-risk PCa are routinely impacted by dependencies on hospital resources and variable procedural wait times ranging from 2 to 8 months. The current study aimed to evaluate whether these extremes of wait times for LDR-BT were associated with worse outcomes for patients with intermediate-risk PCa.

## 2 Materials and methods

### 2.1 Study design & data collection

The electronic medical records for all patients with PCa who received LDR-BT monotherapy at the Tom Baker Cancer Centre in Calgary, Alberta, Canada between 2003 and 2016, were retrospectively reviewed for this study. Data was accessed through centralized provincial electronic health records and electronic medical records systems which house all hospital visitations, diagnostic imaging, pathology, oncology consultation and follow-up and laboratory reports within the jurisdiction. Ethics approval for this study was obtained from the Health Research Ethics Board of Alberta (HREBA).

Predefined inclusion criteria for the current study consisted of patients with NCCN favorable or unfavorable intermediate-risk PCa that were treated with LDR-BT [13]. In brief, all patients had prostate specific antigen (PSA) between 10 and 20 and/or T2c disease and/or Gleason Grade Group 2 or 3 disease and no high risk features [14]. All patients were diagnosed based on trans-rectal ultrasound guided biopsy and all pathology underwent central review by dedicated genitourinary pathologists.

### 3 Treatment characteristics

Prior to LDR-BT, patients underwent gland size assessment on TRUS and CT and patients with gland sizes g50 cc received at minimum 3 months of neoadjuvant androgen deprivation therapy with subcutaneous leuprolide for gland volume reduction. LDR-BT plans were created using the Nucletron SPOT® system (Elekta Inc, Stockholm, Sweden) and all patients were treating using an intraoperatively planned workflow described elsewhere [15]. A 3–5 mm margin was the primary clinical target volume for the prostate gland. Organs at risk including the rectum and urethra were routinely contoured. Institutional dose constraints were: prescribed dose: 144 Gy; D90 prostate > 180 Gy; CTV V100 > 98%; CTV V150 > 70%; CTV V200 > 40%; urethra V140 < 25%; Rectum V100 < 0.3 cc). Inverse planning was used to generate preliminary plans and manual modifications to seed placements within the plan were employed to ensure coverage of biopsy proven disease. Seed activities were 0.346 mCi for glands with volumes < 30 cc and 0.437 mCi for gland volumes > 30 cc.

### 4 Follow-up

Post treatment, patients underwent CT imaging at 1 month to confirm seed placement within the gland. Dosimetry was not routinely calculated at that time and no action ever taken based on undercoverage of any region of the prostate. Patients were instead followed with PSA measurements every 6–12 months for 5 years and then discharged to their family physician with recommendations of yearly PSAs thereafter for life. Patients experiencing recurrent disease were routinely offered hormonal therapy. A small number of young and healthy patients undergoing recurrence received salvage LDR-BT [16].

### 5 Statistical methods

Descriptive statistics were used to describe the cohort. The wait time was calculated as the time from biopsy to LDR-BT procedure. For non-normally distributed continuous variables, medians and inter-quartile-range were used. For binomial and ordinal variables, the absolute numeric count and proportion (percentage) were used. For comparisons of clinical factors between patient favorable and unfavorable intermediate risk cohorts, GraphPAD PRISM software (version 5.02 for Windows, GraphPAD software, San Diego, California, USA) was used. For the cumulative incidence of recurrence (CIR), time to event was defined as time from date of LDR-BT to date of last measured PSA value or recurrent disease on subsequent imaging analysis or biochemical failure (BF) as defined by the Pheonix definition [17]. For the cumulative incidence of metastases (CIM), the time to event was defined as the time from date of LDR-BT to date of metastases on imaging, censured to last medical contact. For OS, the time to event was defined as the time from PCa diagnosis (via biopsy) to death, censured to last medical contact.

Outcomes were analyzed in both univariate models (univariate hazard ratio; UHR) and multivariable models (multivariate hazard ratio; MHR). Univariate modeling included wait time as the sole variable. Multivariable cox proportional sub-distribution hazards models were created for two outcomes: cumulative incidence of recurrence (CIR) and cumulative incidence of metastases (CIM) with competing risks accounted for using Fine and Gray’s method [18]. To determine appropriate covariates to include in multivariate modeling, all variables of interest were analyzed in univariate analyses for P < 0.10 association with the outcomes of interest (Supplemental Table 1). For CIR and CIM, this included patient age (continuous) and the use of androgen deprivation therapy (ADT; yes vs. no). For OS, this included only patient age (continuous), however, ADT use (yes vs. no) was also included as a covariate since this controlled for differences in wait time that occurred due to the use of ADT (which would be an appropriate cause of management delay). Additionally, NCCN risk-group (favourable vs. unfavourable intermediate) was used as a covariate in the analysis of all outcomes when considering all treated patients despite not reaching statistical significance as this is a well-established prognostic factor for outcomes [13] and is a composite variable that controls for differences in measured characteristics listed in Table 1. Non-relapse death was used as a competing risk for CIR and CIM. Two-tailed P-values of < 0.05 were considered statistically significant. All regression analyses were performed using the R programming language version 4.0.0 (www.r-project.org).

**Table 1:** Baseline patient characteristics for the entire cohort and for those with favorable (FIR) and unfavorable (UIR) intermediate-risk prostate cancer. Values given are number (%) or median (Inter-quartile-range) as appropriate.

	All Patients N = 466	FIR PCa N = 296	UIR PCa N = 170	p-value
**Age at Biopsy [years]**	65.1 (59.8–69.3)	64.3 (59.4–69.0)	66.2 (60.4–69.9)	p = 0.039

**Initial PSA [ng/mL]**	7.1 (5.4–9.2)	6.8 (5.3–8.8)	7.5 (5.5–10.0)	p = 0.024

**Wait Time [months]**	5.1 (3.9–6.9)	5.0 (119–208)	5.1 (114–215)	p = 0.765

**Baseline AUA Score**	6 (3–11)	6 (3–10)	6 (3–12)	p = 0.561

**Clinical Stage**				p = 0.137
T1a	1 (0.2%)	1 (0.3%)	0 (0%)	
T1b	0 (0%)	0 (0%)	0 (0%)	
T1c	340 (73%)	224 (76%)	117 (68%)	
T2a	88 (19%)	54 (18%)	34 (20%)	
T2b	28 (6%)	12 (4%)	16 (9%)	
T2c	9 (2%)	5 (2%)	4 (2%)	

**Grade Group**				P < 0.001
1	78 (17%)	61 (21%)	17 (10%)	
2	332 (71%)	235 (79%)	97 (57%)	
3	56 (12%)	0 (0%)	56 (33%)	

**Biopsy Cores Sampled**	11 (10–12)	11 (10–12)	11 (10–12)	p = 0.901

**Biopsy Cores Positive**	4 (2–6)	3 (2–4)	6 (3–7)	P < 0.001

**Biopsy % positive**	8.3 (4.4–14.0)	7.0 (3.4–12.0)	11.0 (7.0–20.0)	P < 0.001

**Perineural invasion**				p = 0.065
Yes	132 (31%)	80 (27%)	63 (37%)	
No	251 (54%)	170 (57%)	81 (48%)	
Unknown	72 (15%)	46 (16%)	26 (15%)	

**Extraprostatic invasion**				p = 1.000
Yes	0 (0%)	0 (0%)	0 (0%)	
No	394 (85%)	250 (84%)	144 (85%)	
Unknown	72 (15%)	46 (16%)	26 (15%)	

**Seminal vesicle invasion**				p = 1.000
Yes	0 (0%)	0 (0%)	0 (0%)	
No	394 (85%)	250 (84%)	144 (85%)	
Unknown	72 (15%)	46 (16%)	26 (15%)	

**Lymphovascular invasion**				p = 1.000
Yes	0 (0%)	0 (0%)	0 (0%)	
No	394 (85%)	250 (84%)	144 (85%)	
Unknown	72 (15%)	46 (16%)	26 (15%)	

**Neoadjuvant ADT**				p = 0.007
Yes	90 (19%)	46 (16%)	44 (26%)	
No	376 (81%)	250 (84%)	126 (74%)	

**Duration of ADT [months]**	3.0 (3.0–3.0)	3.0 (3.0–3.0)	3.0 (1.0–3.0)	p = 0.684

## 6 Results

Four-hundred and sixty-six (466) consecutive patients with a median follow-up of 8.1 years (IQR: 6.3–10.4) met this study’s predefined inclusion criteria. The median wait time for all patients was 5.1 months (IQR: 3.9–6.9). Patient characteristics are displayed in Table 1. Among total patients, the median age at PCa diagnosis was 65.1 years (IQR: 59.8–69.3). 296 (64%) and 170 (36%) patients had favourable and unfavourable intermediate-risk disease, respectively. Dosimetry at the time of LDR-BT is given in Table 2. Median gland volume was 34.3 cc (IQR: 28.3–41.4). Median D90 at time of implantation was 189.9 Gy (IQR: 184.9–193.6) and median CTV V100 was 98.6% (IQR: 97.8–99.3).

**Table 2:** Dosimetry at the time of LDR-BT for the entire cohort and for those with favorable (FIR) and unfavorable (UIR) intermediate-risk prostate cancer. Values given are number (%) or median (Inter-quartile-range) as appropriate.

	All Patients N = 466	FIR PCa N = 296	UIR PCa N = 170	p-value
Activity [mCi]	0.437 (0.436–0.437)	0.437 (0.437–0.437)	0.437 (0.436–0.437)	p = 0.592
Number of Needles	28 (26–30)	28 (26–30)	28 (26–30)	p = 0.452
Number of Seeds	75 (67–84)	74 (67–84)	76 (68–85)	p = 0.389
Gland Volume [cc]	34.3 (28.3–41.1)	34.4 (28.9–40.9)	34.1 (27.2–41.2)	p = 0.487
Prostate D90 [Gy]	189.9 (184.9–193.6)	190.1 (184.9–193.9)	189.4 (184.9–193.2)	p = 0.324
CTV V100 [%]	98.6 (97.8–99.3)	98.6 (97.8–99.3)	98.6 (98.0–99.3)	p = 0.625
CTV V150 [%]	77.8 (75.6–79.8)	77.9 (75.7–80.1)	77.6 (75.6–79.1)	p = 0.122
CTV V200 [%]	44.4 (40.8–47.5)	44.4 (40.5–47.5)	44.6 (41.5–47.7)	p = 0.316
Urethral V150 [%]	0.11 (0.00–0.60)	0.10 (0.00–0.60)	0.18 (0.00–0.58)	p = 0.304
Rectal V100 [cc]	0.04 (0.00–0.15)	0.04 (0.00–0.15)	0.05 (0.00–0.17)	p = 0.401

**Fig. 1:**
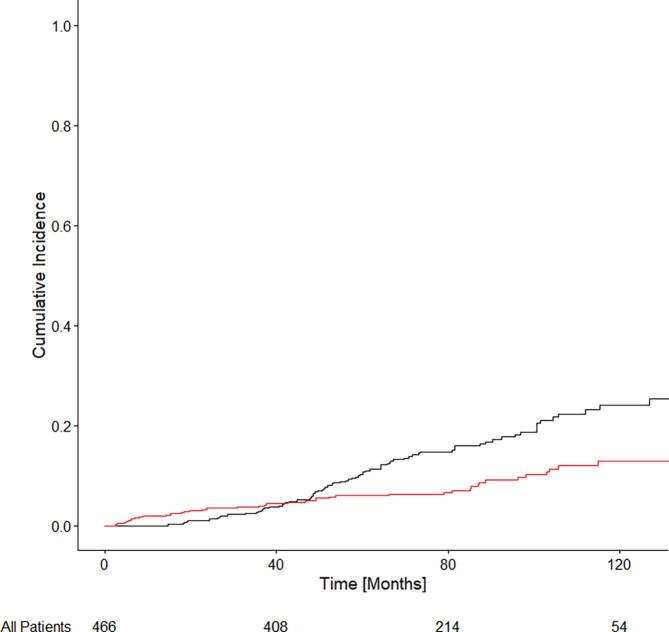
Cumulative incidence of recurrence and competing risk of non-prostate cancer death for all patients with intermediate risk prostate cancer and treated with LDR-BT. The number of patients at risk is shown at the bottom of the figure.

Fig. 1 shows the CIR for the cohort. The estimated cumulative incidence of recurrence at 60 and 120 months were 10.8% (IQR: 10.7–10.8) and 24.0% (IQR: 23.9–24.1), respectively. Amongst all patients, increased wait times for LDR-BT were found to be associated with an increased cumulative incidence of recurrence (CIR) in univariate, but not multivariate models. In univariate modeling, there was a 3% increase in the CIR per month of delay in LDR-BT [UHR = 1.03 (95% CI: 1.01–1.04); p < 0.001] (Supplemental Table 1). In multivariate modeling, there was a trend towards a significant difference in CIR based on wait times [MHR = 1.01 (95% CI: 1.00–1.03); p = 0.080].

When considering only patients with UIR PCa, there was a 3% increase in CIR per month of management delay [UHR = 1.03 (95% CI: 1.02–1.04); p < 0.001]. In multivariate modeling, there was a 1% increase in CIR per month of management delay [MHR = 1.01 (95% CI: 1.00–1.03); p = 0.044]. When considering only patients with FIR PCa there was no statistically significant association found between wait time and CIR on univariate [UHR = 1.02 (0.99–1.06); p = 0.250] or multivariate [MHR = 1.02 (0.97–1.07); p = 0.550] analyses.

**Fig. 2:**
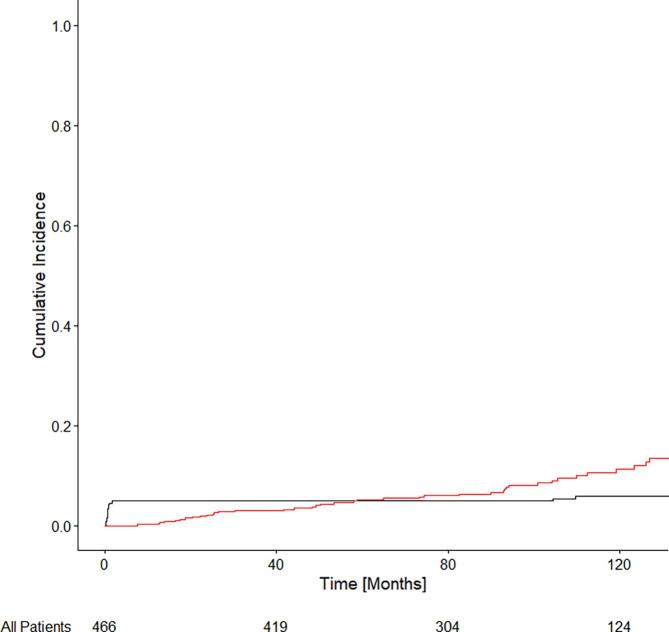
Cumulative incidence of metastatic disease and competing risk of non-prostate cancer death for all patients with intermediate risk prostate cancer and treated with LDR-BT. The number of patients at risk is shown at the bottom of the figure.

Fig. 2 shows the CIM for the Cohort. The estimated cumulative incidence of metastases at 60 and 120 months were 4.9% (IQR: 4.9–4.9) and 6.0% (IQR: 5.9–6.0) respectively. Amongst all patients, increased wait times for LDR-BT were found to be associated with an increased cumulative incidence of metastases (CIM) in both univariate and multivariate models. In univariate modeling, there was a 4% increase in the CIM per month of delay in LDR-BT [UHR = 1.04 (1.03–1.06); p < 0.001]. In multivariate modeling, there was a 3% increase in the CIM per month of delay in LDR-BT [MHR = 1.03 (1.02–1.05); p < 0.001].

When considering only patients with UIR PCa there was a statistically significant association between wait time and CIM in both univariate [UHR = 1.05 (95% CI: 1.04–1.06); p < 0.001] and multivariate [MHR = 1.04 (1.02–1.06); p < 0.001] analyses. When considering only patients with FIR PCa there was no statistically significant association found between wait time and CIM in either univariate [UHR = 1.00 (95% CI: 0.90–1.11); p = 0.940] or multivariate [MHR = 0.99 (0.87–1.12); p = 0.820] analyses.

**Fig. 3:**
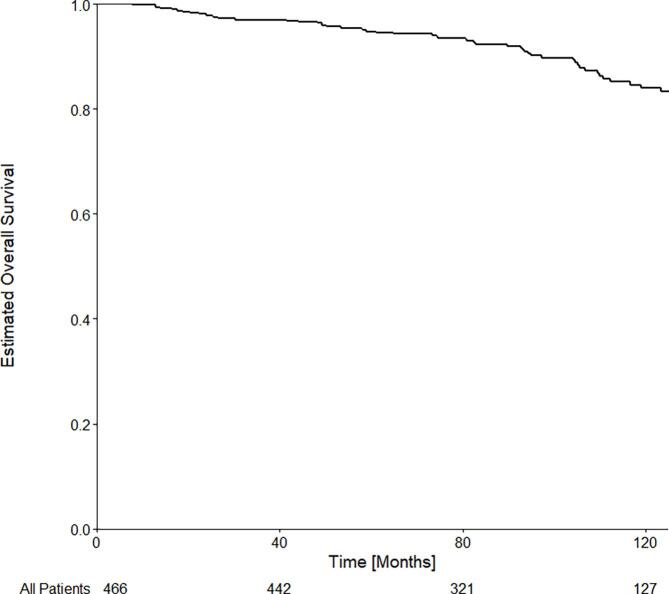
Kaplan-Meier estimated overall survival for all patients with intermediate risk prostate cancer and treated with LDR-BT. The number of patients at risk is shown at the bottom of the figure.

Fig. 3 shows the OS for the Cohort. The estimated overall survival at 60 and 120 months were 94.8% (IQR: 92.7–96.8) and 83.9% (IQR: 79.6–88.5) respectively. Amongst all patients, no statistically significant association was found between increased wait times and overall survival in univariate [UHR = 0.97 (0.93–1.03); p = 0.340] or multivariate [MHR = 0.96 (0.91–1.01); p = 0.126] models.

When considering only patients with UIR PCa there was no statistically significant association between wait time and OS in univariate [UHR = 0.97 (95% CI: 0.89–1.04); p = 0.405] or multivariate [MHR = 0.95 (0.87–1.03); p = 0.220] analysis. When considering only patients with FIR PCa there was no statistically significant association found between wait time and OS in univariate [UHR = 0.99 (95% CI: 0.93–1.05; p = 0.725] or multivariate [MHR = 0.98 (0.92–1.05); p = 0.553] analyses. The complete multivariate analysis is available in Supplemental Table 2.

## 7 Discussion

This analysis of 466 consecutive intermediate-risk PCa patients who underwent LDR-BT at a single comprehensive care cancer center showed that longer wait times were associated with increased CIR and CIM outcomes. For each month of treatment delay, a 1% increased risk in recurrence and a 4% increased risk in metastases was observed in UIR PCa patients. There was no significant association between wait time and OS.

The results of this study are in keeping with those reported by Nam et al for surgical patients in Ontario, Canada. Specifically, they found those patients undergoing radical prostatectomy for prostate cancer and having wait times > 3 months had a 13% decrease in biochemical control when compared to those with shorter wait times [7]. In another study, O’Brien et al found 6 months delays were associated with biochemical progression and surgical upstaging [10]. The two studies that contest these results review experiences where patient wait times were more reasonable (<3 months median) or had shorter follow-up [12], [24]. The present study is unique in that it reports on extensive wait times and is focussed only on patients with intermediate risk disease seeking ablative local treatment. This avoids a possible confounder from including patients whom declined active surveillance for relatively indolent disease [25]. However, it does not differentiate patients with FIR PCa who may be eligible for further disease monitoring [26].

The findings of this study are important given the increased incidence of prostate cancer that is projected moving forward and the potential for a surge in new prostate cancer diagnoses after COVID-19 [2], [19], [20]. Without careful attention to radiotherapy and surgical resource planning, other centers may find expansions of their own waitlists. Despite this, increasing burden of disease may require thoughtful consideration and implementation of appropriate alternatives that are acceptable to patients. In the study center’s anecdotal experience, the primary reason for patients remaining on the LDR-BT waitlist was a lack of acceptable treatment alternatives rather than patient preferences for a delay in treatment. Patients consenting to brachytherapy were often not candidates for radical prostatectomy and for the majority of the study period, the comparable external beam radiotherapy alternative consisted of 78 Gy in 39 daily treatments using 3D conformal radiotherapy planning which presented an alternative with significantly higher risks of gastrointestinal toxicity. Within the study center, to help address the ongoing issues with prostate brachytherapy waitlists the department has introduced a 5 fraction, fiducial guided prostate SBRT program [21], [22], [23]. This is in addition to ongoing advocacy initiatives looking to increase the operating room resources allocated to the prostate brachytherapy program.

This study was subject to several limitations from its retrospective design. Importantly, routine post-implant dosimetry was not calculated on the post-treatment CT images. This may have led to some patients not undergoing appropriate reimplantation for known undercoverage (No patient received reimplantation for undercoverage alone). In centers that routinely reimplant, there is at least theoretically a possibility of lower overall rate of biochemical failure. However, the extensive waitlists experienced over the study period would not accommodate reimplantation procedures. Furthermore, one would not expect the differences seen in failure rates according to wait time to be directly influenced by this practice. Hence if centers that routinely calculate post-implant dosimetry as an actionable event, were to develop such extreme waitlists then the results of this study would likely still be applicable. Additionally, re-staging procedures such as repeat biopsy, repeat digital rectal examination or multiparametric magnetic resonance imaging were not routinely recommended for patients on the waitlist to receive LDR-BT and therefore were not collected for the purposes of this study. Hence it is difficult to determine if there is a discernable clinical upstaging that is directly responsible for the outcomes within this study. However, patients were monitored using PSA blood testing every 2–4 months and over the study period it was common practice to offer external beam radiotherapy to patients with biochemical upstaging. Anecdotally, many patients refused a change in management strategy. These conversations were not recorded reliably enough to meet the scientific standard for inclusion in this study. Finally, although it is the experience of managing physicians at the study center that patients remained on waitlists primarily due to resource constraints that prevented more timely delivery of LDR-BT, the retrospective nature of this study limits the strength of this conclusion. It is possible that other factors such as personal patient preferences for delaying therapy may have contributed to excessive wait times. However, even if this were the case, it is unlikely to have biased the results of this study, as this would required patients with more adverse risk to preferentially opt for a greater delay in treatment, which would not be expected to be the case. Larger multi-center studies would be warranted to more conclusively determine the effect of prolonged wait times on patient outcomes. However, it is likely unethical to impose a deliberate delay in patient treatments for the purposes of such a study.

In conclusion, this study found that increases in wait times for LDR-BT were associated with an increased risk of recurrence and metastases in patients with intermediate-risk PCa. Extensive wait times were a result of resource limitations in the local healthcare system and the results of this study should be considered when planning future resource allocation for genitourinary malignancies in single-payer healthcare systems.

## Declaration of Competing Interest

The authors declare that they have no known competing financial interests or personal relationships that could have appeared to influence the work reported in this paper.
