# Supplementary material for: Excessive waitlists and delays to treatment with low-dose-rate brachytherapy predict an increased risk of recurrence and metastases in intermediate-risk prostatic carcinoma
Source: Clin Transl Radiat Oncol. 2021 Jul 1;30:38–42. doi: 10.1016/j.ctro.2021.06.008 (PMC8283023; doi:10.1016/j.ctro.2021.06.008)
Supplement: Supplementary data 1 [file mmc1.docx]

**Supplemental Content**

**Supplemental Table 1:** Univariable analysis of factors examined for cumulative incidence of recurrence (CIR), cumulative incidence of metastases (CIM) and overall survival (OS) for all patients, patients with favorable intermediate risk prostate cancer (FIR PCa) and patients with unfavorable intermediate risk prostate cancer (UIR PCa).

|  | **All Patients**  **N=466** |
| --- | --- |
| **CIR** |  |
| Wait Time /1m increase | 1.03 (1.01-1.04); p<0.001 |
| Age /1y increase | 1.04 (1.02-1.07); p<0.001 |
| CTV V100 / 1% increase | 1.04 (0.81-1.15); p=0.700 |
| CTV D90 / 1Gy increase | 0.98 (0.95-1.01); p=0.120 |
| NCCN Risk Group UIR vs FIR | 0.95 (0.61-1.48); p=0.820 |
| Neoadjuvant ADT N vs Y | 2.44 (1.54-3.85); p<0.001 |
| **CIM** |  |
| Wait Time /1m increase | 1.04 (1.03-1.06); p<0.001 |
| Age /1y increase | 1.03 (0.99-1.07); p=0.170 |
| CTV V100 / 1% increase | 0.99 (0.68-1.44); p=0.950 |
| CTV D90 / 1Gy increase | 0.97 (0.92-1.03); p=0.300 |
| NCCN Risk Group UIR vs FIR | 1.63 (0.75-3.57); p=0.220 |
| Neoadjuvant ADT N vs Y | 2.99 (1.35-6.62); p=0.007 |
| **OS*** |  |
| Wait Time /1m increase | 0.97 (0.93-1.03); p=0.340 |
| Age /1y increase | 1.05 (1.02-1.08); p=0.004 |
| CTV V100 / 1% increase | 1.24 (0.97-1.61); p=0.091 |
| CTV D90 / 1Gy increase | 1.05 (1.00-1.09); p=0.042 |
| NCCN Risk Group UIR vs FIR | 1.60 (0.96-2.67); p=0.070 |
| Neoadjuvant ADT N vs Y | 1.35 (0.73-2.52); p=0.328 |

*HR are hazards ratio for an increased risk of death

**Supplemental Table 2:** Multivariate analysis of factors examined for cumulative incidence of recurrence (CIR), cumulative incidence of metastases (CIM) and overall survival (OS) for all patients, patients with favorable intermediate risk prostate cancer (FIR PCa) and patients with unfavorable intermediate risk prostate cancer (UIR PCa).

|  | **All Patients**  **N=466** | **FIR PCa**  **N=296** | **UIR PCa**  **N=170** |
| --- | --- | --- | --- |
| **CIR** |  |  |  |
| Wait Time SHR/1m increase | 1.01 (1.00-1.03); p=0.080 | 1.02 (0.97-1.07); p=0.550 | 1.01 (1.00-1.03); p=0.044 |
| Age SHR/1y increase | 1.04 (1.01-1.08); p=0.008 | 1.05 (1.00-1.10); p=0.041 | 1.04 (0.99-1.08); p=0.140 |
| Neoadj ADT SHR N vs Y | 2.24 (1.40-3.58); p<0.001 | 2.25 (1.24-4.07); p=0.007 | 2.14 (0.98-4.67); p=0.055 |
| NCCN Risk SHR UIR vs FIR | 0.79 (0.50-1.26); p=0.320 | NA | NA |
| **CIM** |  |  |  |
| Wait Time SHR/1m increase | 1.03 (1.02-1.05); p<0.001 | 0.99 (0.87-1.12); p=0.820 | 1.04 (1.02-1.06); p<0.001 |
| Age SHR/1y increase | 1.01 (0.98-1.06); p=0.550 | 1.06 (0.98-1.15); p=0.140 | 0.96 (0.90-1.03); p=0.280 |
| Neoadj ADT SHR N vs Y | 2.38 (1.04-5.46); p=0.041 | 1.80 (0.48-6.80); p=0.390 | 3.73 (0.98-14.1); p=0.053 |
| NCCN Risk SHR UIR vs FIR | 1.34 (0.59-3.05); p=0.480 | NA | NA |
| **OS*** |  |  |  |
| Wait Time HR/1m increase | 0.96 (0.91-1.01); p=0.126 | 0.98 (0.92-1.05); p=0.553 | 0.95 (0.87-1.03); p=0.220 |
| Age HR/1y increase | 1.05 (1.02-1.08); p<0.001 | 1.05 (1.00-1.10); p=0.073 | 1.06 (1.02-1.09); p=0.002 |
| Neoadj ADT SHR N vs Y | 1.39 (1.30-1.48); p=0.312 | 1.50 (0.61-3.68); p=0.374 | 1.38 (0.56-3.42); p=0.485 |
| NCCN Risk HR UIR vs FIR | 1.50 (0.90-2.51); p=0.122 | NA | NA |

*HR are hazards ratio for an increased risk of death
